# Type 2 diabetes mellitus control and atherosclerosis prevention in a non-obese rat model using duodenal-jejunal bypass

**DOI:** 10.3892/etm.2014.1832

**Published:** 2014-07-07

**Authors:** XUAN CHEN, ZHEN HUANG, WENHUA RAN, GANG LIAO, LANG ZHA, ZIWEI WANG

**Affiliations:** Department of Gastrointestinal Surgery, The First Affiliated Hospital of Chongqing Medical University, Chongqing 400016, P.R. China

**Keywords:** type 2 diabetes mellitus, duodenal-jejunal bypass, atherosclerosis, c-jun NH_2_-terminal kinase 1, nuclear factor κB

## Abstract

Type 2 diabetes mellitus (T2DM) is a prevalent disease worldwide and during its conventional treatment, vascular complications remain unavoidable. Roux-en-Y gastric bypass (GBP) is able to induce the remission of T2DM. However, studies of duodenal-jejunal bypass (DJB), a modified procedure of GBP, are being carried out to investigate its ability to induce the remission of T2DM and protect the aorta from atherosclerosis. The present study aimed to investigate the effect of DJB on the rate of T2DM remission and the prevention of atherosclerosis in the aorta in rats with streptozotocin-induced diabetes without obesity, and to explore the mechanism of DJB in protecting the aorta from atherosclerosis. A T2DM rat model was established with a high-fat diet and low-dose streptozotocin. Surgery was performed to analyze its effects on glucose homeostasis, lipid metabolism, inflammation and pathological changes. Furthermore, changes in c-jun NH_2_-terminal kinase 1 (JNK1) and inhibitor of κB kinase (IKKβ) genes in the aorta following DJB surgery were examined. Levels of blood glucose, lipids, insulin and tumor necrosis factor (TNF)-α were significantly elevated in the T2DM diabetic model compared with the non-diabetic control. A gradual recovery was observed in the DJB group following surgery. Foam cells and atherosclerotic plaques appeared in the ascending aortic tissue in the sham-surgery and T2DM groups, whereas only slight lesions were observed in the DJB group. The expression levels of JNK1 and IKKβ genes in the aorta were significantly increased in the sham-operated and T2DM groups compared with those in the DJB and normal control groups. The present study demonstrated that DJB caused remission of T2DM without weight loss in non-obese rats. Thus, DJB may delay or prevent the occurrence and development of atherosclerosis in the aorta and this may occur through the JNK1 and nuclear factor κB (NF-κB) signaling pathways.

## Introduction

Type 2 diabetes mellitus (T2DM), which is an endocrine and metabolic disease, has become the third most common type of non-infectious disease worldwide following cardiovascular disease and cancer (1). Conventional treatment of T2DM tends to lead to various complications. Previous studies investigating obesity treatments have demonstrated that Roux-en-Y gastric bypass (GBP) is able to induce the remission of T2DM (2–4), and a further study revealed that GBP is not only able to treat T2DM patients with obesity, but also to prevent the occurrence of complications (5–7). However, whether GBP exhibits the same effect in non-obese patients with T2DM remains unclear. Studies investigating the effects of GBP in non-obese patients with T2DM are limited. Shah *et al* observed that GBP safely and effectively eliminated T2DM in individuals of Asian Indian origin with a body mass index (BMI) <35 kg/m^2^ (8); however, the sample size of the study was only 15 individuals and the follow-up period was only 9 months. Following the examination of a number of studies published in English between 1979 and 2009, Fried *et al* concluded that there were 343 patients with a mean BMI <35 kg/m^2^ who resorted to surgical resolution of T2DM (9). Studies investigating the effects of GBP in T2DM patients with a BMI <30 kg/m^2^ are even more limited.

As a modified GBP procedure, duodenal-jejunal bypass (DJB) does not change the volume of the stomach or restrict food intake as it bypasses the duodenum and upper jejunum. With the aid of Goto-Kakizaki (G-K) rats, Rubino and Marescaux established a spontaneous non-obese model of T2DM, which demonstrated that DJB may be used directly in the treatment of T2DM rather than in weight control or the treatment of obesity (10). However, genetic variations are possible in the spontaneous G-K rat model of T2DM, and G-K rats have a characteristic of inadequate β-cell proliferation that is restricted in the human condition (11). Due to this, and the ability of a high-fat diet (HFD) to induce insulin resistance (IR) and a low dose of streptozotocin (STZ) to cause β-cell damage and thereby induce diabetes mellitus, rats with experimentally induced T2DM were considered to be an appropriate animal model for use the present study and have the potential to illustrate the pathogenesis of human T2DM.

Intensive glycemic control has a protective effect on the microvascular complications of T2DM whereas its effects on macrovascular complications are controversial (12–14). Atherosclerosis is a low-grade subclinical chronic inflammatory disease; the nuclear factor κB (NF-κB) and c-jun NH_2_-terminal kinase (JNK) pathways are two inflammatory signaling pathways that are involved in the occurrence and development of atherosclerosis (15–17). JNK-NF-κB cross communication may play an important role in determining the focal nature of arterial inflammation and atherosclerosis (18). However, studies investigating the protective effect of DJB against atherosclerosis in the aorta are limited.

Thus, the primary objective of the current study was to investigate whether DJB was able to induce the remission of T2DM in non-obese and non-spontaneous diabetic rats. The second objective was to explore the protective effect of DJB against ascending aortic atherosclerosis and analyze the changes in the NF-κB and JNK signaling pathways following DJB surgery in order to identify the possible mechanisms underlying the protective effect. To the best of our knowledge, this is the first study to investigate the protective effect of DJB against atherosclerosis in the aorta.

## Materials and methods

### Materials

Streptozotocin (STZ) was purchased from Sigma, (St. Louis, MO, USA). The insulin (INS) radioimmunoassay kit was purchased from Beijing North Institute of Biological Technology (Beijing, China). The serum triglyceride (TG) and total cholesterol (TC) analysis kits were purchased from Changchun Huili Biological Technology Co., Ltd. (Changchun, China) and the low density lipoprotein (LDL) kit was purchased from Shanghai Rongsheng Biological Pharmaceutical Co., Ltd. (Shanghai, China). The tumor necrosis factor (TNF)-α enzyme-linked immunosorbent assay (ELISA) kit was purchased from Shanghai BiovolBiotech (Shanghai, China). The HFD feed formula contained: 20% fat (50% lard and 50% yolk powder), 20% sugar and 60% regular chow (GB1492413-2001 contains the nutrition standards of regular chow).

### Experimental animals

Four-week-old male Sprague-Dawley (SD) rats (weight, 80–100 g) were acquired from the Experimental Animal Center of Chongqing Medical University, [Chongqing, China; animal license No. SYXK (Chongqing) 2012-0001]. All rats were acclimated to their environment for one week prior to the beginning of the experiment. The rats were housed in standard polypropylene cages and maintained under controlled room temperature (22±2°C) and humidity (55±5%) with a 12/12 h light/dark cycle. The T2DM model group rats received a HFD diet for 4 weeks and were subsequently injected intraperitoneally with low-dose STZ (30 mg/kg) dissolved in citrate solution (0.1 M citric acid and 0.2 M sodium phosphate, pH 4.2–4.5). The rats that were injected with STZ developed diabetes, as indicated by the levels of fasting blood glucose (FBG) being ≥7.8 mmol/l twice or the levels of non-fasting blood glucose being ≥11.1 mmol/l twice during the 4 weeks following the injection (19). The rats with T2DM were randomly divided into three groups: the sham-surgery group (A; n=6) that received a sham surgery; the T2DM control group (B; n=6) that did not undergo surgery and; the DJB group (D; n=6) that underwent DJB surgery. The SD rats of the matched normal control group (C; n=6) received a regular chow diet; rats in groups A, B and D were fed a HFD. The rats were allowed unlimited access to food and water. INS was not administered to any of the animals. The rats were fast for 12–14 h, blood samples were collected in the morning and the levels of FBG, lipids, INS and TNF-α were measured. The rats were sacrificed 12 weeks following DJB surgery to obtain the ascending aortic tissue. The current study was conducted in accordance with the Principles of Laboratory Animal Care (20) for the care and use of experimental animals, and the procedures were approved by the Animal Care Committee of Chongqing Medical University.

### Establishment of DJB and sham-surgery models

The rats were fasted for 12–14 h and anesthetized with 1% sodium pentobarbital (40 mg/kg) via an intraperitoneal injection. For the rats undergoing DJB, the gastric volume was maintained intact while the entire duodenum and the proximal jejunum were bypassed. The stomach was divided from the beginning of the duodenum. A length of 8 cm from the ligament of Treitz was measured to locate the site for the gastrojejunal anastomosis, which was performed using a 6-0 Prolene suture. The continuity of the biliopancreatic secretions was re-established by anastomosing the biliary limb to the alimentary limb of small bowel 12 cm distally to the gastrojejunal anastomosis in a Roux-en-Y fashion (10,21). For the sham surgery, transections and reanastomosis of the gastrointestinal tract were performed at the given sites (corresponding to where enterotomies were performed for the DJB surgery) and the physiological circuit of food was maintained through the bowel. When required, the surgery was prolonged to produce a similar degree of anesthesiological stress to that of the rats who underwent DJB surgery (10).

### Determination of the levels of glucose, lipids, INS, and TNF-α in the blood

Rats were fasted for 12–14 h and blood was collected from the tail vein to analyze the levels of FBG with a glucometer (LifeScan OneTouch^®^; LifeScan, Milpitas, CA, USA). Alternatively, blood was collected from the retro-orbital plexus of the rats under light ether anesthesia using capillary tubes and transferred into Eppendorf tubes. Two methods of blood are mentioned because blood collected from the tail vain is limited and greater amounts of blood are required to separate the serum in order to detect the TC, TG, LDL, and TNF-α levels. The serum was separated by centrifuging at 1,000 × g for 15 min, collected and stored at −80°C. Levels of TC, TG, LDL and TNF-α were measured using commercially available colorimetric diagnostic kits according to the manufacturers’ instructions. The serum INS was assayed by radioimmunoassay according to the manufacturer’s instructions. Homeostatic model assessment-insulin resistance (HOMA-IR) was calculated (HOMA-IR =Fasting blood glucose × Fasting insulin / 22.5) to evaluate insulin sensitivity.

### Histological analysis

Formalin-fixed, paraffin-embedded ascending aortic tissue sections (4 μm) were stained with hematoxylin and eosin for observation under a light microscope (model cx21, Olympus, Tokyo, Japan).

### Quantitative polymerase chain reaction (qPCR)

RNA was extracted from the aortic tissue using RNAiso Plus reagent (Takara Bio, Inc., Shiga, Japan) following the manufacturer’s instructions. Following DNase treatment, RNA was reverse transcribed into cDNA using a PrimeScript^™^ RT reagent kit with gDNA Eraser (Takara Bio, Inc.). Cycling and qPCR detection were performed using a CFX96™ Real-Time PCR Detection system (Bio-Rad, Hercules, CA, USA). The cycling conditions were as follows: 95°C for 30 sec, followed by 39 cycles at 95°C for 5 sec and 60°C for 30 sec. The gene-specific primers were designed using primer analysis software premier version 5.0 (Premier Biosoft, Palo Alto, CA, USA) and the expression of glyceraldehyde 3-phosphate dehydrogenase (GAPDH) was used as the internal control for normalization. The primer pairs used for amplification were: JNK1, 5′-TGGATTTGGAGGAGCGAACTAA-3′ (forward) and 5′-ATTGACAGACGGCGAAGACG-3′ (reverse); inhibitor of κB kinase (IKKβ), 5′-AGTTTGGCATCACATCGGACA-3′ (forward) and 5′-ACCCATCGGGCTCCTCTGTA-3′ (reverse); and GAPDH, 5′-CCGTATCGGACGCCTGGTTA-3′ (forward) and 5′-CCGTGGGTAGAGTCATACTGGAAC-3′ (reverse). Transcription abundance was expressed as fold increase over that of the control gene as calculated by the 2^−ΔΔCt^ method.

### Western blot analysis

Aortic tissue was pulverized into powder in liquid nitrogen and subsequently homogenized in ice-cold modified radioimmunoprecipitation assay (RIPA) buffer. The protein concentration was determined using the bicinchoninic acid (BCA) assay. Equal amounts of protein per lane from each sample were separated on 10% sodium dodecyl sulfate polyacylamide gels and transferred onto polyvinylidene fluoride membranes (Millipore, Billerica, MA, USA). The membranes were incubated with probes overnight using the following antibodies: anti-IKKβ/p-IKKβ (1:1,000 dilution; Abcam, Cambridge, UK), anti-JNK1/p-JNK1 (1:1,000 dilution; Abcam) and anti-GAPDH (1:1,000 dilution; Proteintech, Chicago, IL, USA). The immunolabeled membranes were incubated with goat anti-rabbit immunoglobulin G (IgG) secondary antibodies (1:1,000 dilution; Proteintech) for 2 h. The bands were visualized by enhanced chemiluminescence (Millipore) and quantified using Quantity One software (Bio-Rad, USA).

### Statistical analyses

All results are expressed as mean ± standard error of the mean. Multiple group means were compared by one-way analysis of variance (ANOVA). P<0.05 was considered to indicate a statistically significant difference. SPSS software, version 17.0 (SPSS, Inc., Chicago, IL, USA) was used to carry out the analyses.

## Results

### General results

For the first week following surgery, the body weights of the rats in groups A and D declined (data not shown); however, they exceeded preoperative levels in the second week (although they remained lower than that of the rats in group C; P<0.05) and continued to gradually increase. The body weight increase of the rats in groups A and B was slower than in those of groups C and D following surgery and this difference was significant at 12 weeks (P<0.05).

Three days following STZ injection, the levels of FBG began to increase in the rats with T2DM. The standard for the FBG levels in diabetic models was reached two weeks following STZ injection and continued to increase slowly. By contrast, the levels of FBG in group C rats remained normal (P<0.05).

From the first week following surgery, the levels of FBG markedly decreased in the rats in group D (data not shown), and returned to normal between weeks 2 and 12. However, the levels of FBG in the rats in groups A and B continued to slowly increase (P<0.05).

In the second week following surgery, the levels of TC, TG, LDL, INS and TNF-α, and HOMA-IR in the rats in group D were markedly lower compared with those in the rats in groups A and B (P<0.05). All levels in group D were restored to normal after 12 weeks, with no difference from those in the rats in group C (P>0.05). However, all levels in the rats in groups A and B continued to increase (P<0.05; Table I).

### Pathological analysis

**Figure 1:**
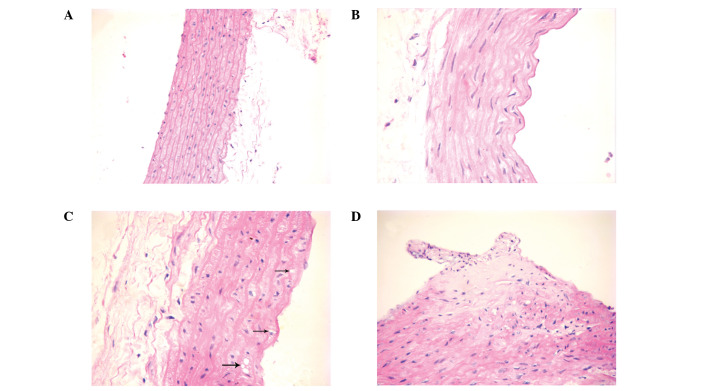
Pathological changes in ascending aortic tissue under hematoxylin and eosin staining. (A) Normal ascending aorta. (B) Arterial tunica intima thickening and protruding into the lumen in rats following duodenal-jejunal bypass. (C) Foam cells (black arrows) and (D) atherosclerotic plaques observed in the aortas of rats with type 2 diabetes mellitus that were untreated or subjected to sham surgery.

A total of 12 weeks following surgery, slight lesions in the ascending aortic tissue appeared in the rats in group D, including arterial tunica intima thickening and the broadening of the subendothelial layer (Fig. 1B). Foam cells appeared in four rats in group A and three rats in group B (Fig. 1C). Atherosclerotic plaques appeared in two rats in group A and three rats in group B (Fig. 1D). The ascending aortic tissue of the rats in group C was normal (Fig. 1A).

### Expression levels of IKKβ and JNK1 mRNA

**Figure 2:**
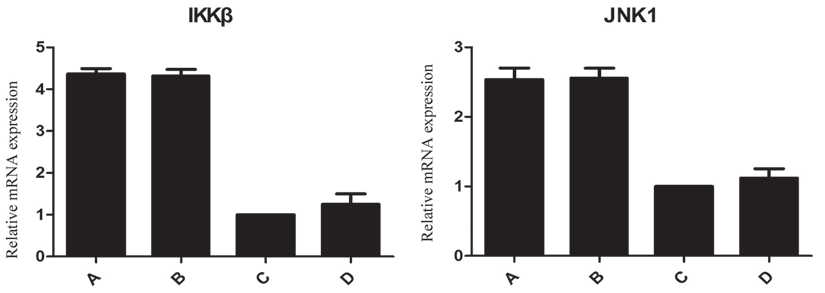
mRNA expression levels of inhibitor of κB kinase (IKKβ) and c-jun NH_2_-terminal kinase 1 (JNK1) in the aorta 12 weeks following surgery were detected by quantitative polymerase chain reaction (qPCR; relative ratio 2^−ΔΔct^) in groups A, B, C and D. The mRNA expression levels of IKKβ and JNK1 in the rats in groups A and B were significantly higher than those in the rats in groups C and D (P<0.05). No difference was observed between these levels in rats in groups C and D (P>0.05). Group A, T2DM model rats subjected to sham surgery; group B, T2DM control; group C, normal control; group D, T2DM model rats subjected to DJB. T2DM, type 2 diabetes mellitus; DJB, duodenal-jejunal bypass.

At 12 weeks following surgery, the mRNA expression levels of IKKβ and JNK1 in the rats of groups A and B were significantly higher compared with those in the rats in groups C and D (P<0.05). No difference was observed in these mRNA levels between the rats in groups C and D (P>0.05; Fig. 2).

### Detection of IKKβ, p-IKKβ, JNK1 and p-JNK1 proteins

**Figure 3:**
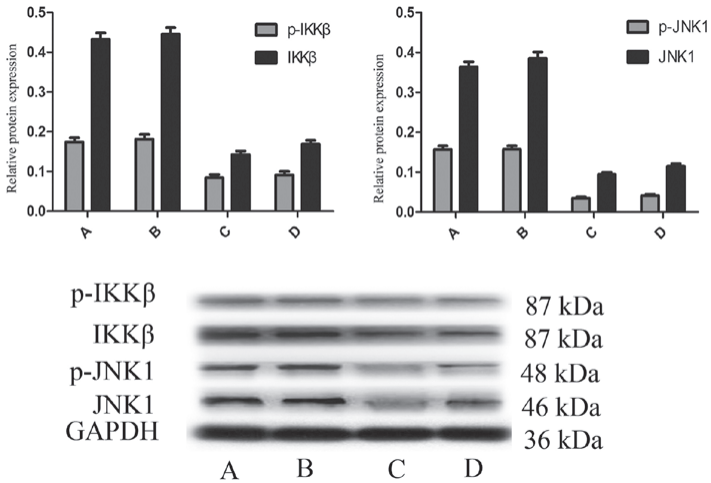
Expression levels of inhibitor of κB kinase (IKKβ)/p-IKKβ and NH_2_-terminal kinase 1 (JNK1)/p-JNK1 proteins in the aorta at 12 weeks following surgery were detected by western blot analysis in groups A, B, C and D. The expression levels of the IKKβ/p-IKKβ and JNK1/p-JNK1 proteins significantly increased in the rats in groups A and B, while they only slightly increased in the rats in group D (P<0.05). There was no statistically significant difference in these levels between the rats in groups C and D (P>0.05). Group A, T2DM model rats subjected to sham surgery; group B, T2DM control; group C, normal control; group D, T2DM model rats subjected to DJB. T2DM, type 2 diabetes mellitus; DJB, duodenal-jejunal bypass.

Western blot analysis revealed that the expression levels of IKKβ, p-IKKβ, JNK1 and p-JNK1 proteins were significantly increased in the rats in groups A and B 12 weeks following surgery, whereas they were only slightly increased in the rats in group D (P<0.05), which was consistent with the qPCR results. No statistically significant difference was identified in the levels of these proteins between the rats in groups C and D (P>0.05; Fig. 3).

## Discussion

GBP may primarily contribute to the reduction of blood glucose levels in patients with T2DM rather than the control of weight (22–24). In severely obese patients with T2DM, GBP surgery has been shown to result in improved glucose control compared with that achieved by medical therapy (25). In a study of obese patients with uncontrolled T2DM, a significantly greater proportion of patients achieved glycemic control through 12-month medical therapy and bariatric surgery compared with medical therapy alone (26). Whether GBP has the same effect on non-obese patients remains unclear. In the present study, the T2DM model rats were non-obese and their FBG levels decreased significantly one week following DJB. As the trauma of surgery affected the rats’ food intake, their body weight slightly declined in the first week. However, two weeks following surgery, the body weights of the rats were restored and even exceeded preoperative levels, and the FBG levels became normal. A total of 12 weeks following surgery, the FBG levels in the DJB-group rats were normal, although their body weights exceeded those of the rats in the sham-surgery and T2DM groups, whose FBG levels continued to increase. The slow increase in body weight in the rats of the sham-surgery and T2DM groups was affected by diabetes. Therefore, it may be concluded that DJB is able to directly control the blood glucose levels of non-obese patients with T2DM without dependence on weight control.

Two weeks following DJB surgery, the levels of FBG in the rats in the DJB group returned to normal; however, the levels of INS remained higher compared with those in the rats in the normal control group, which indicated a significant improvement in IR. This result corresponded with observations made by Pournaras *et al* in patients with T2DM (27), whose study concluded that the increase in INS secretion may be caused by an enhanced glucagon-like peptide 1 (GLP-1) reaction.

Roux-en-Y GBP and biliopancreatic diversion (BPD) are the two most widely used methods of GBP surgery used to treat obesity and T2DM simultaneously in obese patients with T2DM (28–30). As weight loss is not essential in non-obese patients with T2DM, treating T2DM is the direct objective. DJB does not change the volume of the stomach, as it bypasses the duodenum and upper jejunum, and thus it does not restrict food intake. Therefore, in the present study, DJB was adopted in non-obese rats with T2DM in order to investigate whether it was able to induce the remission of T2DM. The results of the current study were consistent with those of Rubino and Marescaux in G-K rats (10), which also demonstrated that the duodenum and upper jejunum may play an important role in the pathogenesis and treatment of T2DM.

JNK may be induced by the endoplasmic reticulum stress triggered by excessive nutrients to cause an inflammatory effect (31). As inflammatory processes play a pivotal role in the pathogenesis of atherosclerosis, anti-inflammatory therapy is expected to become one of the most promising strategies in the treatment of atherosclerosis (32). Further studies have revealed that atherosclerotic processes may be suppressed through the inhibition of the JNK and NF-κB signaling pathways in macrophages and aortic smooth muscle cells (33,34). In the current study, the fact that glucose homeostasis, lipid metabolism and inflammation were improved over time in group D following DJB surgery indicated that the inflammatory effect was decreased. Furthermore, the expression levels of IKKβ and JNK1 mRNA in the aortas of the group D rats were lower compared with those in the rats in groups A and B. Western blotting results revealed that the expression levels of the IKKβ, p-IKKβ, JNK1 and p-JNK1 proteins in the rats in groups A and B significantly increased, while they remained low in the rats in group D with no significant difference from those in the rats in group C. This may be due to the NF-κB and JNK1 inflammatory signaling pathways being activated in groups A and B, but inhibited in group D. Foam cells and atherosclerotic plaques were observed in the aortas of the rats in groups A and B 16 weeks following STZ injection, while only slight lesions appeared in the rats in group D. This indicated that DJB surgery may protect the aorta from atherosclerosis.

In conclusion, DJB was able to induce the remission of T2DM in non-obese rats without weight loss. Furthermore, it delayed or prevented the occurrence and development of atherosclerosis in the aortas of non-obese rats with T2DM. This protective effect was possibly achieved through modulation of the NF-κB and JNK1 signaling pathways.

There were several limitations of the present study. Firstly, the long-term effects of DJB remain unknown as the observation time was limited to 12 weeks. Previous studies have revealed that certain patients with T2DM relapse over a longer follow-up period subsequent to DJB (35,36). Larger, long-term studies are required to confirm the results, and investigation of the cause of relapse may facilitate understanding of the underlying mechanisms and improve the treatment of T2DM.

## Figures and Tables

**Table I:** General results.

Characteristics	Group A	Group B	Group C	Group D
Body weight (g)				
0 week	266.50±8.07	265.33±8.62	264.17±6.40	265.83±7.73
2 week	274.50±8.26^c^	281.00±8.07^c^	306.00±5.44	279.50±7.81^c^
12 week	323.67±9.07^c^	325.83±8.16^c^	441.50±9.20	437.67±9.75^a^,^b^
FBG (mmol/l)				
0 week	16.27±2.46^c^	16.52±2.90^c^	4.43±0.64	16.8±2.27^c^
2 week	17.05±1.64^c^	17.03±2.67^c^	4.40±0.62	5.82±0.25^a^,^b^
12 week	19.58±1.39^c^	20.08±1.10^c^	4.95±0.34	5.13±0.44^a^,^b^
TG (mmol/l)				
0 week	2.47±0.23^c^	2.54±0.25^c^	0.95±0.16	2.50±0.26^c^
2 week	3.12±0.24^c^	3.24±0.23^c^	0.96±0.13	1.69±0.08^a^–^c^
12 week	4.18±0.26^c^	4.12±0.28^c^	1.19±0.15	1.35±0.18^a^,^b^
TC (mmol/l)				
0 week	6.10±0.21^c^	6.13±0.18^c^	3.42±0.23	6.08±0.17^c^
2 week	6.75±0.21^c^	6.71±0.28^c^	3.58±0.31	5.41±0.25^a^–^c^
12 week	8.25±0.29^c^	8.28±0.32^c^	3.90±0.13	4.13±0.38^a^,^b^
LDL (mmol/l)				
0 week	3.44±0.18^c^	3.43±0.24^c^	0.86±0.06	3.42±0.18^c^
2 week	3.72±0.21^c^	3.76±0.19^c^	0.90±0.11	2.47±0.22^a^–^c^
12 week	4.92±0.23^c^	4.89±0.29^c^	1.10±0.18	1.35±0.25^a^,^b^
INS (μIU/ml)				
0 week	12.03±0.49^c^	12.08±0.64^c^	7.74±0.44	12.21±0.51^c^
2 week	14.56±0.89^c^	14.42±0.80^c^	7.85±0.33	12.46±0.70^a^–^c^
12 week	18.75±0.92^c^	18.80±1.25^c^	7.91±0.19	8.22±0.48^a^,^b^
HOMA-IR				
0 week	8.72±1.56^c^	8.88±1.74^c^	1.52±0.22	9.15±1.36^c^
2 week	11.06±1.55^c^	10.90±1.69^c^	1.53±0.19	3.22±0.27^a^–^c^
12 week	16.32±1.41^c^	16.79±1.62^c^	1.74±0.16	1.88±0.26^a^,^b^
TNF-α (pg/ml)				
0 week	138.83±7.55^c^	137.88±9.29^c^	84.67±3.94	138.19±8.11^c^
12 week	174.73±10.57^c^	176.33±9.08^c^	91.98±4.35	100.06±5.87^a^,^b^

a Compared with group A, P<0.05;

b Compared with group B, P<0.05;

c Compared with group C, P<0.05.

Group A, T2DM model rats subjected to sham surgery; group B, T2DM control; group C, normal control; group D, T2DM model rats subjected to DJB. T2DM, type 2 diabetes mellitus; DJB, duodenal-jejunal bypass; FBG, fasting blood glucose; TG, triglyceride; TC, total cholesterol; LDL, low density lipoprotein; INS, insulin; HOMA-IR, homeostatic model assessment-insulin resistance; TNF-α, tumor necrosis factor α.
